# Machine Learning in Palliative Care: Scoping Review of Applications

**DOI:** 10.2196/68317

**Published:** 2026-08-21

**Authors:** Marya Zaidi, Elham Dolatabadi, Peter Tanuseputro, Waqas Ullah Khan, Emily Seto

**Affiliations:** 1 Institute of Health Policy, Management and Evaluation, Dalla Lana School of Public Health, University of Toronto, Health Sciences Building 155 College Street, Suite 425, Toronto, ON, M5T 3M6, Canada, 1 (416) 978-4326, 1 (416) 978-7350; 2 Faculty of Health, School of Health Policy and Management, York University, Toronto, ON, Canada; 3 Vector Institute, Toronto, ON, Canada; 4 Department of Family Medicine and Primary Care, The University of Hong Kong, Hong Kong, China (Hong Kong); 5 Centre for Digital Therapeutics, Toronto General Hospital Research Institute, University Health Network, Toronto, ON, Canada

**Keywords:** machine learning, palliative care, end-of-life care, electronic health records, quality of care, health data analytics, clinical decision support, scoping review, explainable AI, health equity, natural language processing

## Abstract

**Background:**

Palliative care is increasingly recognized as essential for an aging population and rising life-limiting illnesses. Machine learning (ML) has been widely applied in this field, primarily for prognostication. However, recent literature suggests broader applications that may enhance patient-centered care and optimize system-level processes.

**Objective:**

This study aimed to map and summarize the evolving landscape of ML applications in palliative care through a scoping review, identifying how studies extend beyond mortality prediction into new domains, while assessing explainability, equity, and implementation readiness.

**Methods:**

We conducted a scoping review following the Arksey and O’Malley framework and PRISMA-ScR (Preferred Reporting Items for Systematic Reviews and Meta-Analyses extension for Scoping Reviews) guidelines. Six databases (MEDLINE, PsycINFO, Embase, CINAHL, Scopus, and Web of Science) were searched from inception to April 15, 2021, with an update through February 9, 2026. Included studies were peer-reviewed primary studies applying ML to palliative care contexts. Each study was coded for explainable AI (XAI) methods, equity considerations, and implementation readiness. Two reviewers independently screened and extracted data. Synthesis combined descriptive statistics and inductive thematic analysis. Consistent with scoping review methodology, no formal risk-of-bias assessment was performed.

**Results:**

We included 121 studies (2015‐2026) spanning 24 countries, with 69.4% (84/121) published from 2021 onward. The United States contributed the largest share (66/121, 54.5%), followed by Japan, Taiwan, and China (22/121, 18.2%). Cancer was the most commonly studied population (52/121, 43%). Supervised classification was the most common approach (84/121, 69.4%), followed by natural language processing and text mining (16/121, 13.2%). Six application domains were identified: mortality and survival prediction (51/121, 42.1%), health care use (25/121, 20.7%), symptom assessment and phenotyping (20/121, 16.5%), communication and natural language processing (16/121, 13.2%), clinical decision support and care quality (6/121, 5%), and other (3/121, 2.5%). Approximately half of the studies (61/121, 50.4%) used at least one XAI technique, most commonly feature importance rankings and SHAP (Shapley Additive Explanations) values. Among the 121 studies, equity in model performance was fully addressed in only 8 (6.6%) studies, partially in 8 (6.6%) studies, and not addressed in 105 (86.8%) studies. Two-thirds of studies (80/121, 66.1%) remained at the proof-of-concept stage, while 16.5% (20/121) achieved external validation and 17.4% (21/121) reached prospective deployment or clinical integration.

**Conclusions:**

ML applications in palliative care are expanding beyond prognostication toward patient-centered uses, including symptom management, clinical decision support, and resource planning. The persistent gap in equity reporting (105/121, 86.8% did not report equity considerations) signals that the field risks developing tools that may not perform equitably across diverse populations. While half of studies now use XAI techniques, fewer than 1 in 5 studies (21/121, 17.4%) have reached clinical integration. Bridging this translational gap requires systematic attention to implementation science, equity auditing, and explainability reporting.

## Introduction

Palliative care has become a critical component of health systems globally, driven by an aging population and a rise in life-limiting illnesses [1,2]. Managing the trajectory of a life-limiting illness, which includes periods of stability, remission, and eventual decline, poses significant challenges for patients, caregivers, and health care providers alike [3]. Palliative care aims to address the physical, psychological, social, and spiritual needs of these patients, focusing on alleviating suffering and enhancing the quality of life [4]. However, although an estimated 75% of people nearing the end of life (EOL) could benefit from palliative care, access remains limited, with only a minority of eligible individuals receiving it [1,4]. The early integration of palliative care, especially for patients with advanced cancer, has demonstrated clear benefits, including improved quality of life, greater coping ability, and increased willingness to discuss EOL preferences with health care providers [5].

Machine learning (ML), a subset of AI, involves algorithms that learn patterns from data to make predictions or decisions, without requiring explicit rules programmed by humans. The digitalization of health care and the proliferation of structured and unstructured data have fueled interest in applying ML to complex health challenges [6]. Although ML techniques have been in use for decades, their widespread application within health care, particularly in clinical decision-making and patient care, has seen rapid growth primarily in the last decade [7]. Electronic health records (EHRs), as repositories of vast structured and unstructured data, present unique opportunities for ML to support outcome prediction and patient care planning [8]. However, clinical settings introduce specific challenges that complicate the implementation of standard ML methodologies, such as variability in data quality and the ethical implications of automated predictions [9].

Palliative care has attracted growing attention as a target for ML applications, with editorials in the field emphasizing the potential of big data and AI to address the unique challenges in this area [9-12]. Early reviews mapped the nascent evidence base: Storick et al [13] conducted a rapid review through December 2018 and identified only 3 relevant studies using ML on routine data to support EOL care, noting the field was in its infancy. Vu et al [14] expanded this in a systematic review of 22 studies through February 2022, establishing best-practice benchmarks for ML model development but not assessing explainability or equity.

More recently, Bozkurt et al [15] performed a scoping review of 125 studies through December 2023, applying the MI-CLAIM (Minimum Information about Clinical Artificial Intelligence Modeling) framework to evaluate transparency and reporting quality; however, their broader inclusion of gray literature, conference proceedings, dissertations, and knowledge-based systems limits direct comparability with reviews restricted to peer-reviewed ML applications. Migiddorj et al [16] addressed the explainability gap specifically, reviewing 28 palliative care studies through the lens of the CHAMAI (Checklist for Assessment of Medical AI) checklist for explainable AI (XAI), though their narrower scope excluded studies without an explicit XAI component. Despite these contributions, no review has simultaneously assessed explainability methods, equity considerations, and implementation readiness across the full range of ML applications in palliative care.

The aim of this review is to map the scope and characteristics of existing literature on ML applications within palliative care, answering the research question: “What is known in the literature about machine learning applications in the context of palliative care?” In addition to cataloging study designs, populations, and ML methods, we assess explainability practices, equity considerations, and implementation readiness across included studies.

## Methods

### Study Design

The framework of Arksey and O’Malley was used for this scoping review, which consists of 5 stages: identifying the research question; identifying relevant studies; study selection; charting the data; and collating, summarizing, and reporting the results [17]. PRISMA-ScR (Preferred Reporting Items for Systematic Reviews and Meta-Analyses extension for Scoping Reviews) checklist was used to guide the reporting of this review [18,19] (Checklist 1).

### Selection of Sources of Evidence

Titles and abstracts were screened by 2 independent reviewers applying inclusion and exclusion criteria. Full-text screening was also performed independently by 2 reviewers. Any disagreements at either stage were resolved by consulting a senior researcher.

### Eligibility Criteria

We structured our eligibility criteria around a modified Population, Intervention, Comparison, Outcome (PICO) framework to delineate the scope of included studies. Specifically, we defined our target population (P) as patients who would benefit from palliative care, their caregivers, or health care professionals involved in palliative care delivery. Studies not addressing palliative care, that focused on evaluating cohorts based on discrete treatment (eg, chemotherapy and organ transplant) were not included. For the intervention (I), all ML approaches including supervised, unsupervised, and deep learning methods were considered without restricting outcomes (O), as we were interested in identifying the broad range of outcomes in palliative care where an ML approach is applied. A specific comparator (C) was not required, consistent with scoping review methodology aimed at broadly mapping existing evidence rather than evaluating comparative effectiveness.

Selection of publications was limited to English language, involving ML applications for palliative care populations (patients, caregivers, and health care professionals). Studies focusing on evaluating specific treatments (eg, chemotherapy and organ transplant) rather than palliative care services were excluded. Book chapters, lecture notes, conference abstracts without accompanying full text articles, dissertations, and gray literature were excluded. The inclusion and exclusion criteria are provided in Table 1.

**Table 1.** Eligibility criteria for study selection.

Domain	Inclusion criteria	Exclusion criteria
Population	Patients with actual or potential palliative care needs; caregivers; or health care professionals involved in palliative, end-of-life care delivery.	Studies unrelated to palliative care or end-of-life care; not focused on human subjects.
Intervention and approach	Peer-reviewed primary studies applying ML^a^ techniques (supervised, unsupervised, deep learning, and NLP^b^) within palliative or end-of-life care contexts.	Studies focused solely on discrete disease treatments (eg, chemotherapy and organ transplant); without a palliative care context; methodological papers on ML methods without an applied palliative use case.
Outcomes	Any outcome relevant to palliative care (eg, mortality, survival, use, symptoms, processes, quality, communication, and phenotyping).	—^c^
Study type	Original research (retrospective, prospective cohorts, trials, and pilot, feasibility with primary data).	Reviews, protocols, editorials, commentaries, book chapters, dissertations; conference abstracts without accompanying full-text peer-reviewed articles; gray literature.
Language and availability	English; full text available	Non-English publications, unavailable full text

a ML: machine learning.

b NLP: natural language processing.

c Not applicable.

### Identifying Relevant Studies

A systematic search was conducted using 6 electronic databases: MEDLINE, PsycINFO, Embase, CINAHL, Scopus, and Web of Science. Initial searches covered publications from inception to April 15, 2021, with an updated search through February 9, 2026. The results were imported into EndNote and Covidence where the duplicates were removed.

### Search Strategy

The search keywords targeting the main search concepts of “machine learning” and “palliative care” were developed based on the literature. Recommended search terms from the palliative care literature [20] were incorporated to develop a structured search strategy for MEDLINE and adopted for the subsequent databases. This search strategy was subsequently reviewed and refined through consultation with a librarian at the University of Toronto. The keywords are provided in Table 2, and an example of the search strategy is provided in Multimedia Appendix 1.

Because the original search (inception to April 15, 2021) and the updated search (2021 to February 9, 2026) were conducted at different time points using the same databases and search terms, the study selection process is reported as a dual-stream PRISMA (Preferred Reporting Items for Systematic Reviews and Meta-Analyses) flow diagram consistent with PRISMA 2020 guidance for updated reviews [19]. Studies identified in both streams (n=6) [21-26], arising from the overlap between search windows during the first 3.5 months of 2021, were counted once in the final total.

**Table 2.** Main concepts and related keywords.

Concept	Matching keywords
Palliative care	Palliative medicine, palliative therapy, terminal care, end-of-life care, hospice, bereave, end stage, terminally ill, last year of life
Machine learning	Machine learning, artificial intelligence, data mining, deep learning, neural networks

### Data Charting

A data charting form was drafted by the authors using Microsoft Excel spreadsheet software to systematically abstract relevant characteristics from the included studies. Extracted information comprised study metadata, including authors, year of publication, country of study, study design; population details, such as the study setting, main outcomes measured, and sample size; and ML methodologies, covering algorithms used, tools or software, performance measures, and outputs (if available). Consistent with standard scoping review methodology, a formal risk-of-bias assessment or quality appraisal of individual studies was not conducted.

In addition, each study was assessed on 3 supplementary dimensions. First, XAI was coded as present if the study used at least one post hoc or intrinsic interpretability method (eg, SHAP [Shapley Additive Explanations], LIME [Local Interpretable Model-Agnostic Explanations], feature importance ranking, attention visualization, or counterfactual explanation) or used an inherently interpretable model as the primary approach (eg, logistic regression, decision tree, or topic model with reported coefficients or rules).

Second, equity considerations were coded on a 3-level scale: “yes” if the study explicitly reported model performance stratified by demographic characteristics (eg, race, ethnicity, sex, age, or socioeconomic status), applied bias mitigation strategies, or reported formal equity metrics; “partial” if the study identified or discussed demographic disparities in its findings without formally stratifying model accuracy or discrimination metrics by subgroup; and “no” if equity, or demographic performance differences were not addressed.

Third, implementation readiness was classified using a 3-tier framework: Tier A, model development with internal validation only (eg, train-test split, cross-validation on a single institutional dataset); Tier B, external validation or multisite testing on data not used during model development; and Tier C, prospective deployment, real-time clinical integration, or evaluation embedded within a clinical workflow (eg, pragmatic trial, alert-based intervention, or clinician-facing decision support tool evaluated in practice).

Coding criteria for all 3 dimensions were developed iteratively; initial categories were pilot-tested on a subset of 15 studies, after which the classification scheme was refined and condensed into the final categories reported here. The primary reviewer applied the finalized coding guide across all included studies, while a second reviewer independently verified the coding, with disagreements resolved through discussion.

### Synthesis of Results

Extracted data were summarized descriptively (frequencies and percentages). The reviewers inductively coded and extracted the data manually, with full-text verification as needed, using Microsoft Excel to organize extracted data and iteratively refine themes. Themes were developed to categorize key areas of ML application in palliative care into meaningful groups. No formal interrater statistic was calculated; agreement was reached through discussion, and final themes were validated by cross-checking a subset of studies. These thematic categories were then used to interpret the current scope of ML applications in palliative care and identify key implications for future research, clinical practice, and policy. Visual summaries (graphs and figures) were created to support this interpretation. Figures were created programmatically using Python 3.10 (Python Software Foundation).

## Results

### Included Articles

**Figure 1.**
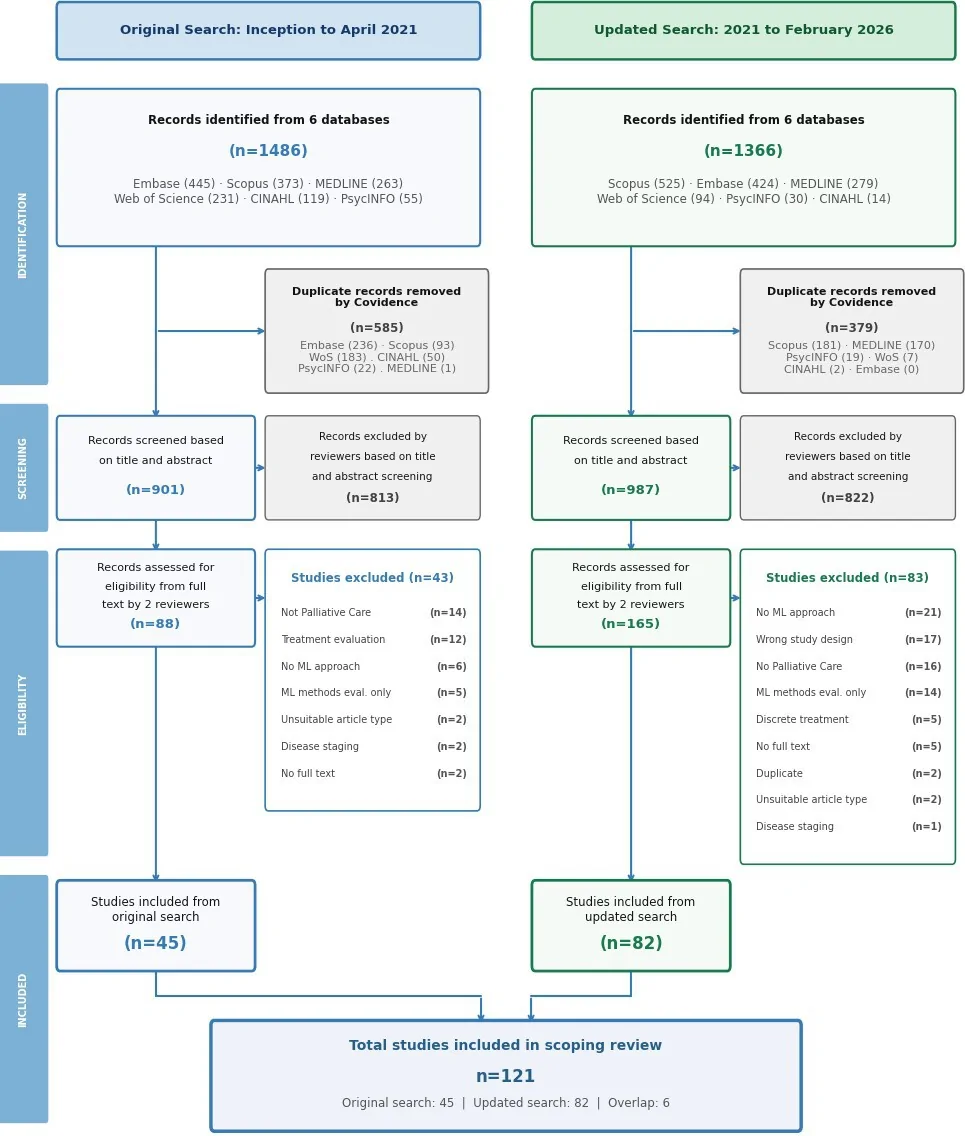
PRISMA flow diagram showing study selection process. ML: machine learning.

A total of 121 unique studies [21-141] were included in this review. The original search (inception to April 15, 2021) identified 1486 records across 6 databases, of which 45 studies met inclusion criteria after deduplication and screening [21-26,30,31,33-36,39-41,50,52,55,56,65,70,71,73,75,77,85,86,89,92,96-100,106,108,109,119,120,126,128,130-133]. The updated search (April 16, 2021 to February 9, 2026) identified 1366 additional records, yielding 82 eligible studies [21-29,32,34,37,38,42-49,51,53,54,57-64,66-69,72,74,76,78-84,87,88,90,91,93-95,102-105,107,110-118,121-125,127,129,134-141]. After removing 6 studies [21-26] that appeared in both searches, 121 unique studies [21-141] were included in this review (Figure 1).

### Characteristics of Included Articles

**Figure 2.**
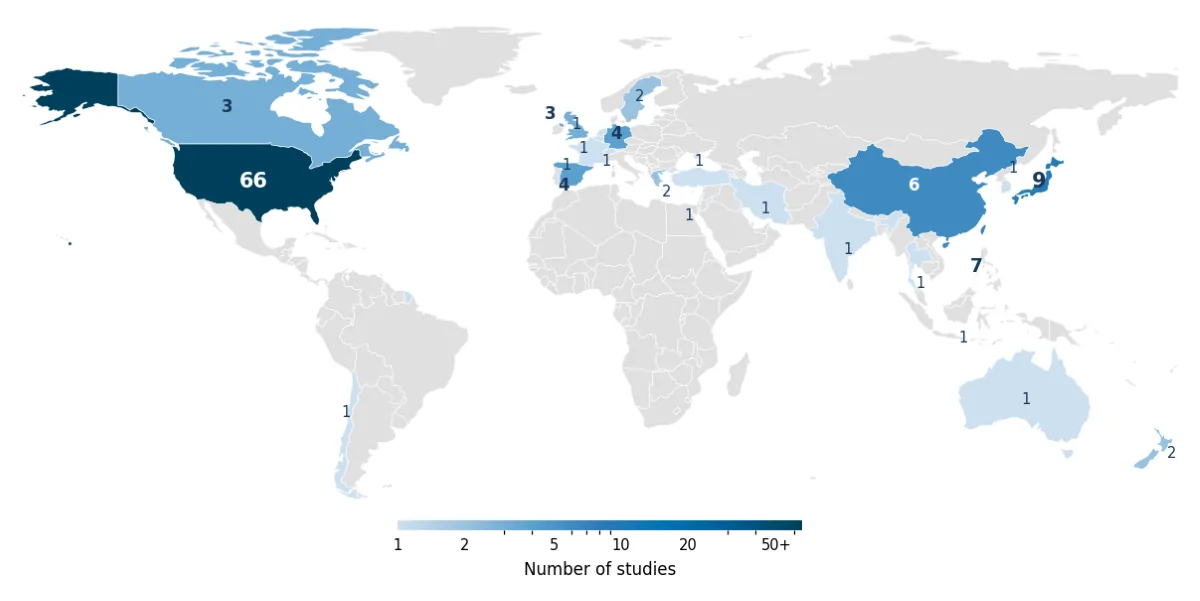
Geographic distribution of included studies (N=121).

Included studies spanned the period 2015 to 2026, with 84 [21-29,32,34,37,38,42-49,51,53,54,57-64,66-69,72,74,76,78-84,87,88,90,91,93-95,101-105,107,110-118,121-127,129,134-141] of 121 [21-141] (69.4%) studies published from 2021 onward, reflecting the rapid growth of this field. Sample sizes varied substantially, ranging from feasibility studies with fewer than 100 participants (n=7) [28,38,72,80,91,112,137] to large-scale analyses of datasets exceeding 100,000 records (n=20) [29,33,34,36,39,52,55,57,69,71,75,82,83,97,98,107,116,118,120,128], with the majority falling between 100 and 99,999 (Table 3). The United States contributed the largest share of studies (66/121, 54.5%) [21,23-26,28-30,32,33,36,40,43,46,49,53,55-61,65,66,68,71,73-75,78,80,84,87,92-95,97-101,105,107-111,113,115,116,118-120,124,127,128,130-135,139,140], followed by Japan (n=9) [54,70,76,81,103,104,106,123,136], Taiwan (n=7) [44,72,85,89-91,137], China (n=6) [45,62,63,86,114,138], Spain (n=4) [22,37,38,96], and Germany (n=4) [48,51,64,122]; in total, 24 countries were represented (Figure 2).

**Table 3.** Characteristics of included studies (N=121)^a^.

Characteristic	Studies, n (%)
Overview^b^	
Total studies included	121 (100)
Studies published from 2021 onward	84 (69.4)
Countries represented (n=24)	
United States	66 (54.5)
Japan	9 (7.4)
Taiwan	7 (5.8)
China	6 (5.0)
Spain	4 (3.3)
Germany	4 (3.3)
Other (18 countries)	25 (20.7)
Study design	
Retrospective cohort	92 (76.0)
Prospective cohort	12 (9.9)
RCT^c^ and interventional	7 (5.8)
Cross-sectional	4 (3.3)
Other	4 (3.3)
Qualitative and mixed methods	2 (1.7)
Sample size	
<100	7 (5.8)
100‐999	33 (27.3)
1000‐9999	29 (24.0)
10,000‐99,999	30 (24.8)
≥100,000	20 (16.5)
Not reported	2 (1.7)
Disease focus	
Cancer	52 (43.0)
Not disease-specific	45 (37.2)
Multidisease	9 (7.4)
Dementia and ADRD^d^	5 (4.1)
COPD^e^	3 (2.5)
Other^f^	7 (5.8)
Setting	
Inpatient	49 (40.5)
Mixed and multisetting	35 (28.9)
Outpatient	20 (16.5)
Hospice and home-based PC^g^	9 (7.4)
ED^h^	4 (3.3)
Community	4 (3.3)
Primary outcome domain	
Mortality and survival prediction	51 (42.1)
Health care use	25 (20.7)
Symptom assessment and phenotyping	20 (16.5)
Communication and NLP^i^	16 (13.2)
Clinical decision support and care quality	6 (5.0)
Other	3 (2.5)
ML^j^ task category	
Supervised classification	84 (69.4)
NLP and text mining	16 (13.2)
Deep learning	6 (5.0)
Regression and survival analysis	4 (3.3)
Unsupervised clustering	4 (3.3)
Time-series	4 (3.3)
Other	3 (2.5)
Prediction time horizon	
In-hospital or real time	14 (11.6)
≤30 days	15 (12.4)
31‐180 days	15 (12.4)
1 year	18 (14.9)
>1 year	3 (2.5)
Multihorizon composite	15 (12.4)
Not time-bound or not applicable	41 (33.9)
Primary data source	
EHR^k^, structured data	51 (42.1)
EHR with clinical notes and NLP	23 (19.0)
Multimodal, mixed sources	14 (11.6)
Administrative and claims data	12 (9.9)
Other and nonclinical	8 (6.6)
Patient-reported outcomes	6 (5.0)
Wearable and sensor data	4 (3.3)
Registry or database	3 (2.5)
Explainability (XAI^l^)	
At least one XAI method reported	61 (50.4)
No XAI reported	60 (49.6)
Equity reporting	
Studies with any equity-relevant analysis	16 (13.2)
Implementation readiness	
Tier A: Model development, internal validation only	80 (66.1)
Tier B: External validation or multi-site testing	20 (16.5)
Tier C: Prospective or clinical workflow integration	21 (17.4)

a Values are presented as n (%) unless otherwise specified.

b Publication year range=2015‐2026.

c RCT: randomized controlled trial.

d ADRD: Alzheimer disease and related dementias.

e COPD: chronic obstructive pulmonary disease.

f Other disease focus includes COVID-19 (n=2), liver disease, cirrhosis (n=2), renal disease (n=1), hip fracture (n=1), and cardiac arrest with anoxic brain injury (n=1).

g PC: palliative care.

h ED: emergency department.

i NLP: natural language processing.

j ML: machine learning.

k EHR: electronic health record.

l XAI: explainable AI.

### Study Design, Population, and Sample Size

The majority of studies used retrospective cohort design (92/121, 76%) [21-26,28-37,39,40,42-44,46,48-53,55,56,58,61-67,69-71,73,75-79,82-85,87-90,92,95,96,99,102-108,110,111,113-117,119,120,122-134,136,138,140,141], reflecting the predominant use of existing clinical databases and EHRs for model development. A smaller subset used prospective cohort designs (12/121, 9.9%) [27,57,59,68,72,81,91,97,98,112,137,139] or interventional approaches including randomized controlled trials and controlled pre-post studies (7/121, 5.8%) [47,60,74,94,100,101,135]. Cross-sectional studies (4/121, 3.3%) [45,54,118,121] and studies using other or mixed designs (4/121, 3.3%) [41,86,93,109] accounted for the remainder, with 2 (1.7%) studies [38,80] using qualitative or mixed methods frameworks to evaluate ML tools in palliative care contexts. Table 3 presents aggregate characteristics of included studies.

The disease focus of included studies was concentrated in 2 dominant categories. Cancer populations represented the largest share (52/121, 43%) [28,31,40-42,48,51,53,58-63,67,68,70,72,74,76-81,86,88,90,91,100,101,103,104,106,109-114,116,117,122-125,130,136-139,141], spanning general oncology, metastatic solid tumors, and site-specific cancers including breast, lung, colorectal, and pancreatic malignancies. Studies that did not target a specific disease accounted for 45/121 (37.2%) studies [21,22,24,25,29,32-39,44,45,47,49,50,52,55,57,64-66,71,82-85,87,92-95,97,98,107,115,119,120,127-129,131,135]; these typically drew on general hospital, emergency department, or community-dwelling populations and used administrative or EHR data without restricting to a particular diagnosis. Nine (7.4%) studies [30,54,56,69,99,118,121,134,140] enrolled multidisease cohorts that included combinations of cancer, heart failure, chronic obstructive pulmonary disease (COPD), and dementia. Disease-specific studies outside oncology were markedly less common: dementia and Alzheimer disease–related dementias (5/121, 4.1%) [102,105,126,132,133], COPD (3/121, 2.5%) [26,27,73], liver disease (2/121, 1.7%) [75,89], COVID-19 (2/121, 1.7%) [43,108], and single studies each addressed end-stage renal disease, hip fracture, and cardiac arrest with anoxic brain injury. The pronounced concentration of disease-specific studies in cancer populations, alongside the relative absence of models developed for heart failure, COPD, renal disease, and dementia, represents a notable gap given that these conditions are among the leading drivers of palliative care need globally.

Most studies drew on inpatient or hospital-based populations (49/121, 40.5%) [22,23,25,29-31,33,35,37,46,47,50,53,56,62-66,68,72,73,79,82-85,87,89,90,92,94-98,102,103,105,107,108,113-115,127,134-137], with an additional 35 (28.9%) studies [21,24,26,32,38,40,43,45,48,52,55,70,71,75-78,81,86,88,91,99,100,104,106,109,112,119,120,122,123,130-133] spanning mixed or multisetting contexts. Fewer studies were conducted in outpatient settings (20/121, 16.5%) [28,41,42,51,57-61,67,74,80,101,110,111,117,118,124,139,141], hospice or home-based palliative care (9/121, 7.4%) [44,49,54,93,121,125,126,128,138], community settings (4/121, 3.3%) [27,36,129,140], or emergency departments (4/121, 3.3%) [34,39,69,116]. Clinical setting categories are summarized in Table 3.

Sample sizes varied widely, ranging from small feasibility samples to population-level datasets exceeding 2.7 million individuals (Table 3). Aggregate characteristics of included studies across all of these dimensions are summarized in Table 3.

### Primary Outcomes

Outcomes were heterogeneous across the 121 included studies [21-141], reflecting the clinical breadth of palliative care practice. Within the mortality domain (51/121, 42.1%) [21,26,27,29-31,33,35,36,39,43,48,50,51,61,70,72,73,75,82,86,89,91,93,96-99,101,102,105,106,108,109,111,114-116,119,120,122,124,128,129,133,136-139,141], operationalization varied substantially. Prediction windows ranged from in-hospital death within hours of a clinical event to population-scale 15-month prognostication, with 6-month and 1-year horizons most commonly used. Several studies used composite end points combining mortality with hospice enrollment or discharge status, and a small number used the clinician surprise question as a surrogate mortality marker rather than a recorded death end point. Health care use outcomes (25/121, 20.7%) [22,23,25,32,34,37,44,52,55,57,58,65-69,71,76,78,88,90,125,127,135,140] were predominantly framed as clinical action triggers—time-to-palliative care consultation, hospice delivery model selection, avoidable emergency visit prevention, and demand forecasting for palliative services—rather than purely predictive targets.

**Figure 3.**
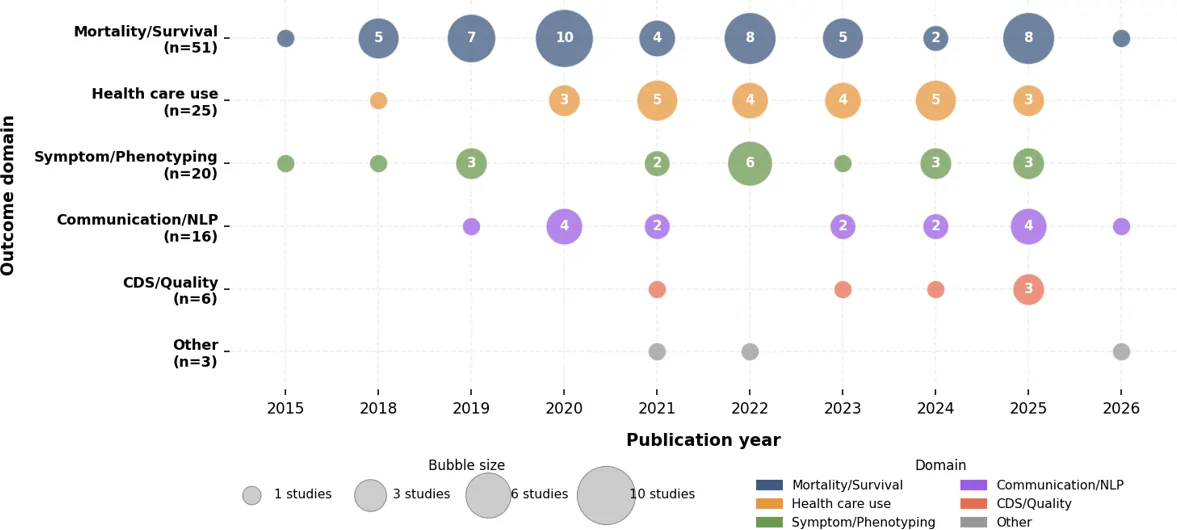
Evidence gap map of machine learning applications in palliative care by publication year and primary outcome domain (N=121; bubble size proportional to study count). CDS: Clinical decision support; NLP: natural language processing.

Symptom and phenotyping outcomes (20/121, 16.5%) [41,49,53,54,56,62,64,74,79,81,85,87,92,103,104,110,117,121,123,132] were divided into 2 methodologically distinct groups: studies using validated instruments such as the Brief Pain Inventory, the Generalized Anxiety Disorder-7 (GAD-7), and the Delirium Rating Scale-Revised-98 (DRS-R98), and an emerging group extracting symptom signals from clinical notes and voice recordings using natural language processing (NLP) and speech recognition methods. Communication and NLP studies (16/121, 13.2%) [24,28,40,42,59,60,77,83,84,94,100,107,113,130,131,134] measured process outcomes—rates of goals-of-care documentation, advance care planning conversation completion, and serious illness discussion identification in clinical records—rather than direct patient physiological end points. Clinical decision support studies (6/121, 5%) [38,47,63,80,95,118] assessed implementation-oriented outcomes including clinician perceptions of ML tools, model usability, and equity of deployed deterioration algorithms. The distribution of primary outcome domains across the study period is shown in Figure 3.

### Prediction Time Horizons

Prediction timeframes varied considerably across the 80 studies [22,23,25-27,29-31,33,34,36-39,43,47-53,55,57-62,65-70,72,73,75,81-85,91,92,94,95,97-103,105-111,113-116,118-120,124,127-129,133,135-141] with time-bound prediction horizons. The most common window was 1 year or longer, reported in 21 (26.3% of time-bound studies) studies [22,27,36-38,48,55,57,59-61,67,82,102,115,118,119,128,133,140,141]; within this group, 18 (22.5%) studies [22,27,36-38,48,55,57,61,67,82,102,115,118,119,128,140,141] used a 1-year horizon specifically and 3 (3.8%) studies [59,60,133] used horizons beyond 1 year. This concentration reflects the predominance of mortality and survival prediction in the literature. Medium-range horizons of 31 to 180 days were used by 15 (18.8%) studies [29,30,33,99-101,105,107,109-111,114,120,124,139], predictions within 30 days of admission, discharge, or a clinical event were used by 15 (18.8%) studies [25,31,34,39,52,58,65,68,69,83,84,91,97,98,135], and a further 15 (18.8%) studies [23,26,43,51,66,70,75,92,94,96,106,113,129,136,138] used multihorizon composite designs evaluating multiple simultaneous timeframes spanning hours to 5 years. The remaining 14 (17.5%) studies [47,49,50,53,62,72,73,81,85,103,108,116,127,137] generated in-hospital or real-time predictions. A separate 41 (33.9% of all included studies) studies [21,24,28,32,35,40-42,44-46,54,56,63,64,71,74,76-80,86-90,93,95,104,112,117,121-123,125,126,130-132,134] were not time-bound, reflecting NLP, phenotyping, clustering, and descriptive applications that classified existing data rather than predicting a future event within a specified window.

### ML Approaches

ML techniques were categorized according to standard definitions commonly used in literature: supervised learning (models trained with labeled data), unsupervised learning (models identifying patterns from unlabeled data), and deep learning (complex neural network architectures) [7]. Supervised classification was by far the predominant ML approach (84/121, 69.4%) [21-23,27,29-31,33,34,36-39,44-52,55,57-61,63-69,71,73,75-77,79,81,85,86,89-102,104,106-109,111-116,118-124,126,128,136-141], followed by NLP and text mining (16/121, 13.2%) [24,28,40-42,53,54,56,83,84,103,105,117,130,131,134], deep learning architectures (6/121, 5%) [43,62,72,82,127,133], regression and survival analysis (4/121, 3.3%) [26,35,70,129], unsupervised learning and clustering (4/121, 3.3%) [ 32,78,87,132], time-series methods (4/121, 3.3%) [25,110,125,135], and other approaches (3/121, 2.5%) [74,80,88].

Studies used between 1 and 9 distinct algorithms. Within supervised classification, the most commonly used algorithms included logistic regression (reported in 47 studies [21,23,27,29,31,36,37,39,44-49,51,52,57,61-63,65,68,69,71,73,75,79,90-93,99,102-104,109,113,115,118-122,126,128,140,141]), random forests (n=45) [21,22,24,30,39,44-46,48,50,52,53,57,63,65,68-71,73,79,82,89-91,95-99,102-104,108,109,115,119-122,126,129,138,140,141], support vector machines (n=25) [22,26,30,36,39,41,44,45,69,79,85,89,91,92,99,102-104,118,122,124,126,138,140,141], gradient boosting variants including XGBoost (Extreme Gradient Boosting) and LightGBM (n=36) [22,30,32,34,36-39,44,52,63,68,75,76,79,89,91,103,104,107,109-111,113,116,118,122,124,126,129,135,136,139-141], and decision trees (n=14) [30,36,44,45,48,81,85,89,90,92,102,121,123,128]. Deep learning architectures, which appeared across multiple task categories, encompassed recurrent neural networks including Long Short-Term Memory models (n=14) [21,26,35,41,43,67,72,82,86,96,125,127,133,137], convolutional neural networks, and multilayer perceptrons. NLP-specific methods ranged from traditional approaches such as latent Dirichlet allocation and bag-of-words models to transformer-based architectures including BERT (Bidirectional Encoder Representations from Transformers). Four studies (3.3%) [28,42,84,105,117] applied large language models, specifically GPT-4o, Llama 3.3, and Phi-3, primarily for goals-of-care documentation, clinical text classification, and symptom detection from unstructured notes. Ten studies reported ensemble strategies combining multiple algorithms, including voting classifiers, stacking, and gradient-boosted ensembles. Additional approaches included Bayesian methods, causal inference frameworks, Cox proportional hazards regression, and comparison frameworks for mortality prediction.

### Explainability and Interpretability

**Figure 4.**
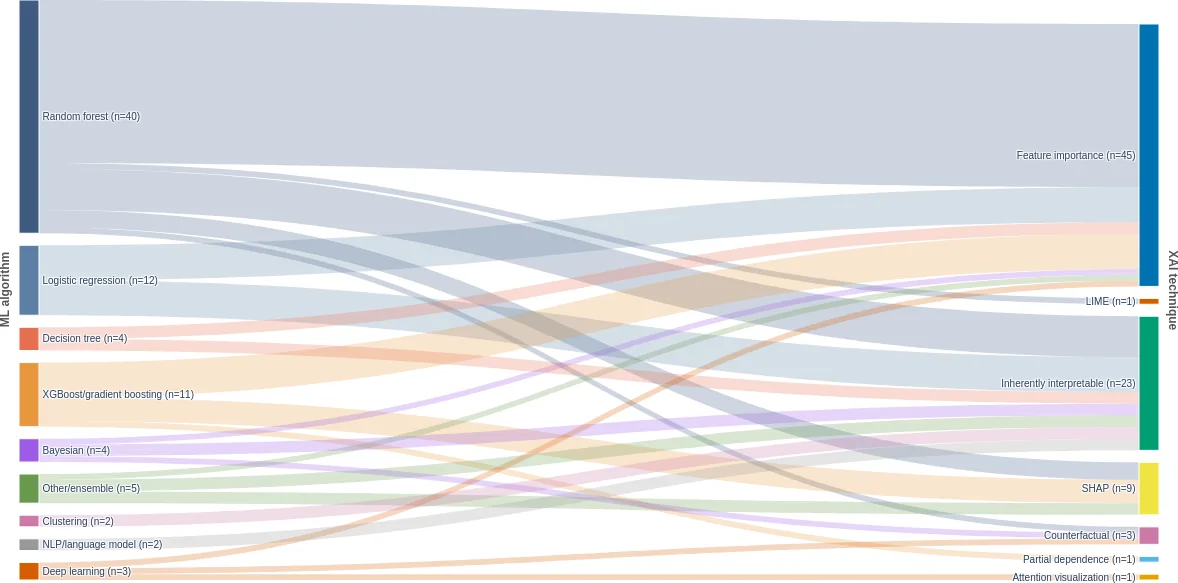
Explainability methods by machine learning algorithm among studies reporting at least one explainable AI technique (n=61; flow width proportional to number of studies). ML: machine learning; LIME: Local Interpretable Model-Agnostic Explanations; NLP: natural language processing; SHAP: Shapley Additive Explanations; XAI: explainable artificial intelligence.

Of the 121 included studies [21-141], 61 (50.4%) studies [22,24,25,27,29,30,32,33,36-39,44-46,48-50,52,56,63,64,67-70,73,75-79,81,83-85,87-92,95,96,99,104,107-109,116,119,120,122-124,128,129,133,135,136,141] used at least one XAI method. The relationship between ML algorithm type and XAI method used across the 61 studies reporting explainability is illustrated in Figure 4. Each flow connects an ML algorithm family on the left to the XAI techniques it was paired with on the right, with flow width proportional to the number of studies.

The most frequently used approach was feature importance rankings (n=45), followed by SHAP (n=9), counterfactual explanations (n=3), partial dependence plots (n=1), attention-based visualization (n=1), and LIME (n=1). An additional 23 studies [27,29,36,39,48,49,52,56,64,68,70,73,77,78,81,83,84,87,88,92,95,123,128] within the XAI reporting group used inherently interpretable models such as logistic regression, decision trees, or topic models as their primary or comparative approach. Many studies used more than one interpretability technique. A further 11 studies [23,26,40,51,102,105,112,117,130-132] used inherently interpretable models without explicitly framing this as an explainability approach. The remaining 60 (49.6%) studies [21,23,26,28,31,34,35,40-43,47,51,53-55,57-62,65,66,71,72,74,80,82,86,93,94,97,98,100-103,105,106,110-115,117,118,121,125-127,130-132,134,137-140] did not report any interpretability technique.

### Equity Reporting

Equity considerations were notably absent from the majority of the literature. Only 8 (6.6%) studies [29,43,47,57,66-68,93] explicitly reported equity in model performance through subgroup performance by race, ethnicity, sex, or socioeconomic status, or applied bias mitigation strategies (Table 4). An additional 8 (6.6%) studies [32,60,61,78,92,97,98,116] partially addressed equity by acknowledging potential disparities without formal evaluation. The remaining studies (105/121, 86.8%) made no mention of equity in their model development or evaluation.

**Table 4.** Equity-related analyses conducted across included machine learning studies (n=16).

Study	Equity tier	Equity assessment
Agarwal et al (2022) [29]	Full	C-statistics were reported separately by race and sex in the temporal validation cohort. Race was excluded from the model as a bias-mitigation strategy, and formal fairness metrics were calculated.
Chi et al (2022) [43]	Full	Model calibration and discrimination were assessed separately by race in supplementary materials, and fairness metrics were reported.
Colacci et al (2025) [47]	Full	Sensitivity and specificity were stratified across 5 sociodemographic subgroups: age, sex, homelessness, neighborhood socioeconomic status, and neighborhood racialized composition. A formal fairness evaluation was conducted.
Frechman et al (2025) [57]	Full	AUC^a^, sensitivity, PPV^b^, equal opportunity, and equalized odds were reported separately by sex, race, ethnicity, and age, constituting a full algorithmic fairness audit.
Handler et al (2023) [66]	Full	AUC-PR^c^ was reported separately by race and ethnicity, sex, socioeconomic status (ADI^d^), and rurality, representing a formal equity evaluation across 5 demographic dimensions.
He et al (2024) [67]	Full	Model performance was stratified by rural-urban location, immigration status, and world region of birth, and formal fairness metrics were reported.
Herskovits et al (2024) [68]	Full	Model AUC was reported separately by race and language group.
Lu et al (2022) [93]	Full	A comprehensive fairness audit was conducted, including AUC, calibration, sensitivity, specificity, PPV, and FPR^e^ reported separately by sex and race and ethnicity across 3 clinical settings, with intersectional subgroup analysis.
Aude et al (2025) [32]	Partial	Clustering analysis identified demographic subgroups by race, sex, and age, and differences in palliative care consultation timing were examined across groups. Equity implications were discussed, but no model performance metrics were stratified by demographic subgroup.
Gensheimer et al (2025) [60]	Partial	Race was excluded as a predictor as a bias-mitigation strategy. Equity implications were discussed, but no subgroup performance metrics were reported.
Gensheimer et al (2025) [61]	Partial	Race and ethnicity were excluded as predictors, and equity implications were discussed, including comparison with a vendor model. No demographic subgroup performance evaluation was conducted.
Khayal et al (2023) [78]	Partial	Clustering analysis identified racial disparities in hospice use across 362 hospitals. Race was a central analytic variable with substantive discussion of systemic inequities, but no model performance metrics were stratified by demographic subgroup.
Lodhi et al (2015) [92]	Partial	Model accuracy was reported across 4 age strata (young, middle-aged, old, and very old). No analysis was conducted for race, ethnicity, or other protected characteristics, and no fairness framing was provided.
Major and Aphinyanaphongs (2020) [97]	Partial	Demographic differences across hospital sites were described, and model performance variation was noted across sites with differing racial compositions. Equity implications were discussed, but no formal subgroup performance metrics were reported.
Major et al (2020) [98]	Partial	This deployment-focused companion study discussed real-world performance variation across sites and ethical considerations for model governance. No subgroup performance metrics were reported.
Qiao et al (2022) [116]	Partial	AUC was reported separately by age group, sex, income quartile, and cancer type. No fairness metrics, bias analysis, or interpretation of subgroup disparities was conducted.

a AUC: area under the curve.

b PPV: positive predictive value.

c AUC-PR: area under the precision-recall curve.

d ADI: Area Deprivation Index.

e FPR: false positive rate.

### Implementation Readiness

Regarding implementation readiness, the majority of studies (80/121, 66.1%) [21,23,24,26-28,31,32,35-38,40-42,44,45,48-50,53-56,61,63-65,70-73,75-78,80,81,83-88,90-92,95,96,99,102-104,106,108,110,112,115-121,123-134,136-138,141] were classified as Tier A, reflecting model development with internal validation only. Twenty (16.5%) studies [29,30,34,39,46,51,52,57,62,66,67,69,82,89,93,105,111,114,122,139] achieved Tier B through external validation or multisite testing, and 21 (17.4%) studies [22,25,33,43,47,58-60,68,74,79,94,97,98,100,101,107,109,113,135,140] reached Tier C, involving prospective deployment, real-time clinical integration, or workflow-embedded evaluation.

### Thematic Groups

#### Overview

To help answer the research question regarding what is known in the literature about areas of ML applications in the context of palliative care, the results of this scoping review are presented in 6 thematic application domains. These domains correspond to the primary outcome classification, though individual studies occasionally addressed objectives spanning more than one theme; in such cases, the study was assigned to the domain reflecting its principal reported outcome.

#### ML for Predicting Mortality, Survival, and EOL Outcomes

This remains the most extensively studied application of ML in palliative care, encompassing models that predict short-term and long-term survival to guide care planning and timely palliative interventions. Studies in this category ranged from hospital-level 30-day mortality prediction to population-scale 12- and 15-month prognostication in Medicare beneficiaries, and included disease-specific models for advanced cancer, dementia, and metastatic bone disease. Supervised classification and tree-based algorithms were the predominant approaches [29,39,72,97], with recent studies extending mortality prediction to residential aged care [129], pancreatic cancer [122], and multiparametric survival modeling in advanced oncology [69,108].

The long-term impact of ML-triggered behavioral nudges on serious illness conversations and EOL outcomes has also been evaluated in a randomized clinical trial [101], and wearable actigraphy-based survival estimation has been explored as a novel data modality [91,137].

#### ML for Predicting Health Care Use and Resource Allocation

Studies in this category focused on forecasting hospital admissions, readmissions, emergency department visits, and the need for palliative or hospice services to improve resource allocation and proactive care. Applications included identification of patients likely to benefit from specialist palliative care referral [21,125,140], prediction of hospice enrollment [44], early palliative intervention for high-risk groups such as hip fracture patients [23], and clinical text mining for palliative care need identification [88]. Additional studies predicted hospital readmissions and palliative services needs to enable timely interventions [32,52,65,71], and demographic disparities in palliative care consultation timing have been examined using clustering approaches [32]. Mortality prediction at hospital admission has been used to guide palliative care program enrollment [22], and real-world deployment of risk-based prescriptive analytics to reduce avoidable emergency visits [58]. Predictive modeling has also been embedded into hospital systems to improve palliative care consultation delivery [25], and trigger AI-assisted palliative care referrals through a randomized clinical trial [135]. This domain had among the highest rates of prospective clinical deployment among all thematic groups, with 6 studies integrated into real-world workflows [22,25,58,68,135,140].

#### ML for Symptom Assessment, Phenotyping, and Patient Profiling

This domain, which has grown substantially compared with earlier reviews, includes studies applying classification, clustering, and feature extraction methods to characterize patient subgroups based on symptom burden, disease trajectories, patient-reported outcomes, and functional decline. Unsupervised learning has been used to identify EOL care intensity trajectories in pediatric populations [87] and patient clusters based on mortality risk trajectories and associated use patterns at EOL [110]. Additional studies have developed complementary frailty and mortality prediction models to identify distinct palliative care phenotypes among older adults [37] and applied EHR-based ML models for proactive phenotyping of patients with advanced oncology conditions [141]. NLP-based methods have extracted symptom information from clinical notes, including detection of social distress, spiritual pain, and severe symptoms [103,123], and voice recognition combined with ML has been tested for detecting patient-reported outcomes from conversational speech [54]. A conversational agent for collecting patient-reported quality-of-life outcomes was also explored as a feasibility study [41]. Additional studies addressed prediction of specific complications, including delirium [79,81], pressure injuries [85], pain [92], and anxiety in palliative settings [64]. Emerging applications include AI systems for detecting psychospiritual distress in family caregivers [104] and small language models for uncovering symptom burden from unscheduled visits [117].

#### AI and NLP for Communication and Clinical Documentation

Studies in this theme leveraged NLP, large language models, and text classification to support patient-provider communication, identify serious illness conversations in clinical documentation, and extract goals-of-care information from EHRs. NLP and deep learning models have been developed to identify serious illness conversations [130], goals-of-care documentation [24,131], and advance care planning discussions in clinical notes [28]. This area has expanded notably with the emergence of large language models, with zero-shot large language models applied to measure documented goals-of-care discussions [84], and ML-triggered lay care coach programs have increased advance care planning conversations for patients with metastatic cancer [59,60]. Topic modeling and sentiment analysis of clinician-patient conversations [42] and NLP-based detection of goals-of-care documentation using active learning [134] represent further diversification. Place-of-death prediction using causal exploration methods [77] and word embedding analyses of EOL language [83] illustrate additional applications within this domain. Six studies in this domain reached prospective clinical deployment, including ML-triggered advance care planning programs [59,60,94,100,107,113].

#### Improving Palliative Care Processes Through ML Implementation

This domain encompasses ML applications designed to enhance palliative care processes by embedding predictive models into clinical workflows. Parikh et al [109] developed and validated a 6-month mortality prediction algorithm that was subsequently embedded into oncology workflows to trigger serious illness conversations. User-centered design of clinical decision support systems for palliative care has been explored [38], and ML-based tools have been evaluated for predicting palliative care phases to guide clinical management [63]. Additional studies examined clinician perceptions of mortality prediction tools for identifying palliative care needs [80,118], equity evaluations of deployed deterioration models [47], and cross-field attribute embedding for clinical endpoint prediction [86]. Reliability and equity considerations in predictive models for advance care planning were systematically evaluated by Lu et al [93]. ML has also been used to transform standardized nursing care plan data into meaningful clinical variables for palliative care research [95].

#### Other Applications

A small number of studies fell outside the 5 primary domains, including a framework for evaluating ML techniques to predict palliative care decision-making in Alzheimer disease [126], a study using ML to predict behavioral intentions of hospice and palliative care providers [45], and large-scale automated phenotyping of cardiac arrest and withdrawal of life-sustaining therapy (WLST) using EHR data [46]. Unlike the patient-level phenotyping in Theme 3, this study applied ML to identify and classify clinical events rather than characterize individual patient trajectories. Notably, Clive et al [46] modeled WLST as a clinician-driven end point distinct from biological death, using an enriched cohort design and admission-to-death timestamps as a proxy for WLST timing; cross-site performance degradation was observed, illustrating how site-specific documentation practices shape ML model transportability.

Taken together, these 6 thematic domains reflect the breadth and growing diversity of ML applications in palliative care, spanning from survival prediction to real-world clinical implementation.

## Discussion

### Summary of Findings

This scoping review mapped the landscape of ML in palliative care across 121 studies [21-141] published between 2015 and 2026, making this the largest and most comprehensive synthesis in this field to date. Mortality prediction remains the single largest outcome domain, accounting for 42.1% of included studies (51/121) [21,26,27,29-31,33,35,36,39,43,48,50,51,61,70,72,73,75,82,86,89,91,96-99,101,102,105,106,108,109,111,112,114-116,119,120,122,124,128,129,133,136-139,141], and is frequently used as a proxy to identify patients who might benefit from palliative services [33,98]. By estimating death risk at defined time horizons, ML aims to prompt timely palliative interventions, optimize care planning, and potentially reduce burdensome EOL treatments.

At the same time, concentrating solely on mortality has inherent limitations. Patients with serious illness can have substantial palliative care needs even if they are not at immediate risk of death. Studies caution that relying only on mortality risk may exclude individuals who would benefit from palliative care despite longer prognoses [37,121]. To address this, researchers have suggested incorporating additional predictors—such as symptom burden, functional decline, and quality-of-life indicators—into ML models to support more holistic care planning [54,91,121]. Indeed, several recently identified studies have begun integrating such indicators into ML models to address diverse palliative care challenges—for example, predicting complications such as delirium [79], optimizing resource planning for home-based palliative care [125], and supporting clinical decision-making via automated referral prompts for palliative consultations [25,135].

Beyond mortality prediction, ML is being increasingly applied to a broader range of clinically meaningful outcomes. Studies included in this review also focused on forecasting health care use outcomes such as unplanned hospital admissions, readmissions, and other morbidity-related complications, which significantly impact patient quality of life and health care resource use [71,125]. Additionally, recent studies developed ML-based interventions specifically targeting timely palliative care referrals through clinical decision support, advance care planning documentation through NLP, and caregiver support applications. The emergence of symptom assessment, phenotyping, and communication applications alongside these health care use–focused studies indicates meaningful diversification beyond the field’s historically prognostication-centered focus. Clinical decision support studies, though fewer in number, represent a critical translational bridge between model development and care delivery.

### Comparison With Prior Reviews

The trajectory of review evidence in this field illustrates the rapid maturation of ML applications in palliative care. Storick et al [13] conducted a rapid review of 7 databases through December 2018 and identified only 3 studies that applied ML to routine data for EOL care improvement, concluding that the evidence base was nascent and that the field required dedicated investment before clinical translation could be considered. Within 4 years, Vu et al [14] expanded this landscape through a systematic review of 22 studies, establishing important methodological benchmarks by evaluating ML studies against best-practice criteria for model development, validation, and reporting. Their analysis highlighted that many early studies suffered from inadequate validation and poor methodological transparency, setting the stage for more rigorous assessments.

The most directly comparable effort is the scoping review by Bozkurt et al [15], which aimed to systematically map the landscape of AI applications in palliative and hospice care, focusing on three key domains: (1) the purposes and data sources of AI models, (2) the methods and extent of model validation and generalizability, and (3) the degree of transparency and reproducibility [15]. Their review identified 125 studies published until December 2023 and evaluated data transparency and reporting completeness, finding that 86% of included studies were retrospective proof-of-concept designs and that none adhered to AI-specific reporting guidelines. However, several methodological differences between their review and ours warrant consideration. Bozkurt et al [15] adopted a broader inclusion strategy that encompassed gray literature, conference proceedings, and dissertations alongside peer-reviewed journal articles, and defined artificial intelligence to include knowledge-based systems such as rule-based NLP and expert systems in addition to data-driven ML approaches. Our review, by contrast, was restricted to peer-reviewed primary studies applying data-driven ML techniques, which may account for the comparable study counts (125 vs 121) despite our search extending 26 months further through February 2026. Importantly, while Bozkurt et al [15] focused on reporting domains such as reporting transparency and validation rigor, they did not assess whether studies used explainable methods, address equity considerations, or evaluate implementation readiness.

The explainability dimension was specifically addressed by Migiddorj et al [16], who conducted a systematic review to assess how ML models used in palliative care comply with the principles of XAI, guided by the CHAMAI checklist, and addressed 2 research questions: how well current ML models align with XAI principles, and which specific methods are used to enhance model explainability. Their review included 28 palliative care studies. Of these, only 11 (39%) used any explainability technique, and none used advanced approaches such as concept-based, attention-based, or latent-based methods. Although their lower proportion partly reflects their narrower eligibility criteria, which required an explicit XAI component, is the broad direction is consistent with our observation that approximately half of studies (61/121, 50.4%) used at least one XAI technique. Those same criteria, however, precluded assessment of the larger body of work that does not report interpretability practices. Migiddorj et al [16] did not assess equity considerations or implementation readiness.

Our review extends the contributions of these prior syntheses in 3 main respects. First, our search window through February 2026 captures the most recent period of accelerated growth, during which 84 [21-29,32,34,37,38,42-49,51,53,54,57-64,66-69,72,74,76,78-84,87,88,90,91,93-95,101-105,107,110-118,121-127,129,134-141] of 121 (69.4%) included studies [21-141] were published. Second, we are the first to simultaneously assess explainability, equity, and implementation readiness across the full spectrum of ML applications in palliative care, revealing that while approximately half of studies now use some form of model interpretability, the majority still fall short on equity reporting (86.8% did not address equity) and remain at the proof-of-concept stage (66.1% Tier A). Third, our thematic analysis demonstrates that the field has diversified considerably, with symptom assessment, phenotyping, and communication applications together accounting for 29.8% of the literature, compared with the predominantly mortality-focused landscape described in earlier reviews.

### ML Applications Beyond Mortality Prediction

The heavy focus on mortality prediction as a proxy for palliative care needs highlights both the promise and challenges of ML in this space. While mortality risk can help identify patients who might benefit from palliative care, using mortality as a primary criterion may inadvertently overlook patients who could benefit from earlier interventions. Several tools currently exist for prognostication, yet studies indicate that health care providers tend to overestimate prognosis, often delaying palliative care initiation. Here, ML techniques—particularly risk prediction models—show potential to improve prognostic accuracy, reduce uncertainty in clinical decision-making, and facilitate early palliative interventions [33,101,133]. Future studies could address how incorporating nonmortality indicators could enhance ML models in palliative care and broaden the patient populations reached by these tools.

Beyond prognostication, ML has shown promise in supporting quality of care measures and EOL processes. NLP and other ML approaches have shown promise in identifying care process indicators, such as goals-of-care discussions and care preference documentation, which play a crucial role in aligning medical care with patient preferences and improving patient-centered outcomes at the EOL [9,10]. By automating the identification of these indicators, ML can support quality assessments and highlight areas where EOL processes may need enhancement, promoting alignment between clinical actions and patient values [9,10].

However, current studies focusing on these indicators remain limited, and more research is needed to evaluate how ML can be integrated into routine care workflows. Embedding ML-driven triggers for palliative consultations or discharge planning within EHR workflows, for example, could guide clinicians in real-time and streamline palliative care service delivery [97,135]. Recent studies exploring clinical decision support systems and AI-driven conversational models further demonstrate ML’s potential to facilitate communication and patient-centered care delivery in palliative settings [38,49].

### Recommendations for Future Research and Practice

First, studies should prioritize rigorous validation and patient-centered evaluation. The wide range of ML algorithms and sample sizes across included studies makes cross-study comparison challenging. Future work should emphasize sufficient sample sizes, standardized performance metrics, and robust validation techniques including external and prospective designs. Comparative studies across different ML architectures, especially deep learning and ensemble models, may provide insights into optimal approaches for specific palliative care applications. Critically, model performance should be evaluated on patient-centered outcomes including quality of life, symptom management, and patient satisfaction, rather than relying solely on technical metrics such as accuracy and area under the curve [13,21,141].

Second, equity auditing should become standard practice. Only 16 [29,32,43,47,57,60,61,66-68,78,92,93,97,98,116] of 121 (13.2%) studies [21-141] addressed equity considerations in any capacity, representing a critical gap. As ML tools are increasingly deployed in clinical settings serving diverse patient populations, future studies must evaluate model performance across racial, ethnic, and socioeconomic subgroups and report calibration metrics alongside discrimination metrics. Models should be developed transparently, validated across diverse patient groups, and implemented with sensitivity to patient autonomy, dignity, and individualized care [13,21,141].

Third, the field should invest in implementation science to bridge the translational gap. Fewer than 1 in 5 studies in this review progressed to prospective or clinically integrated evaluation. Moving ML tools from development to deployment requires not only technical validation but also attention to workflow integration, clinician trust, and organizational readiness. Future research should prioritize pragmatic trials that evaluate the real-world impact of ML-assisted palliative care interventions on patient outcomes and clinician decision-making. The shift toward virtual and home-based palliative care also presents opportunities for ML approaches that support remote monitoring and telehealth-enabled interventions.

Fourth, reporting standards and methodological transparency require improvement. Studies should adopt AI-specific reporting guidelines, share code and data where feasible, and ensure transparency and respect for patient autonomy in model development. The treatment of withdrawal of life-sustaining therapy as a mortality-equivalent outcome in EHR-based studies also warrants closer methodological scrutiny; future work should report WLST prevalence, cohort assembly strategies, and site-specific validation results separately from biological mortality endpoints.

### Limitations

This scoping review has limitations inherent to the methodology and scope. First, our search strategy excluded gray literature, conference proceedings, and dissertations, which may have omitted relevant unpublished studies. Second, because we restricted inclusion to English-language publications, the review is subject to language bias and may have excluded relevant studies published in other languages. Third, consistent with scoping review methodology, we did not perform formal quality appraisal or risk-of-bias assessment. Fourth, while our implementation tier framework captured the level of validation achieved, we did not formally assess methodological characteristics such as overfitting risk or sample size adequacy. These factors could affect generalizability and clinical applicability. Fifth, the XAI, equity, and implementation tier assessments were coded by the primary reviewer with independent verification by a second reviewer using the coding guide described in the “Methods” section. While explicit criteria were applied and disagreements were resolved through discussion, this approach may introduce classification subjectivity compared with fully independent dual coding. Finally, the implementation tier system used in this review is a simplified 3-level framework and does not capture the full spectrum of translational readiness that more granular frameworks might provide.

### Conclusions

This scoping review mapped 121 studies (2015‐2026) [21-141] on ML in palliative care, revealing a field that has grown rapidly, with over two-thirds of studies published since 2021. Mortality prediction remains the dominant application (51/121, 42.1%), yet the evidence base is diversifying toward health care use, symptom assessment, communication analysis, and clinical decision support. Approximately half of included studies (61/121, 50.4%) now use at least one XAI technique, suggesting growing but incomplete adoption of model transparency; however, nearly half of all studies still report no interpretability method. Equity remains the most significant gap: 86.8% (105/121) of studies did not address equity, and only 13.2% (16/121) conducted equity-related analysis. Implementation readiness remains limited, with two-thirds of studies at the proof-of-concept stage and only 17.4% (21/121) reaching prospective deployment or clinical integration. To realize the potential of ML in palliative care, future research must prioritize equity assessment, external validation, prospective evaluation, and integration into clinical workflows, alongside transparent reporting of model limitations and performance across diverse populations.

## Supplementary material

10.2196/68317 Multimedia Appendix 1 Sample search strategy.

10.2196/68317 Checklist 1 PRISMA-ScR checklist.

## Abbreviations

BERT: Bidirectional Encoder Representations from Transformers
CHAMAI: Checklist for Assessment of Medical AI
COPD: chronic obstructive pulmonary disease
DRS-R98: Delirium Rating Scale-Revised-98
EHR: electronic health record
EOL: end of life
GAD-7: Generalized Anxiety Disorder-7
LIME: Local Interpretable Model-Agnostic Explanations
MI-CLAIM: Minimum Information about Clinical Artificial Intelligence Modeling
ML: machine learning
NLP: natural language processing
PICO: Population, Intervention, Comparison, Outcome
PRISMA: Preferred Reporting Items for Systematic Reviews and Meta-Analyses
PRISMA-ScR: Preferred Reporting Items for Systematic Reviews and Meta-Analyses extension for Scoping Reviews
SHAP: Shapley Additive Explanations
WLST: withdrawal of life-sustaining therapy
XAI: explainable AI
XGBoost: Extreme Gradient Boosting
