# Supplementary material for: Machine Learning in Palliative Care: Scoping Review of Applications
Source: JMIR AI. 2026 Aug 21;5:e68317. doi: 10.2196/68317 (PMC13496941; doi:10.2196/68317)
Supplement: Supplementary file 2 [file ai-v5-e68317-s001.docx]

**Multimedia Appendix 1. Sample Search Strategy**

**Ovid MEDLINE**:

Epub Ahead of Print, In-Process & Other Non-Indexed Citations, Ovid MEDLINE® Daily and Ovid MEDLINE® <1946-Present>

1. exp Palliative Care/
2. exp Terminal Care/
3. exp Terminally Ill/
4. exp Hospices/
5. ("end-of-life" or "end of life" or "end-stage" or "end stage" or "last year of life" or bereav* or Palliat*).ti,ab,kw.
6. 1 or 2	or 3 or 4 or 5	226,882
7. exp Machine Learning/	25,912
8. ("data mining" or "artificial intelligence" or "machine learning" or "deep learning" or "neural net*").ti,ab,kw.	115,823
9. 7 or 8	123,851
10. 6 and 9	263

**Embase Classic + Embase:** <1947 to 2021 April 13>

1. exp Palliative Care/ or exp Terminal Care/ or exp Terminally Ill/ or exp Hospices/ 184265

2. ("end-of-life" or "end of life" or "end-stage" or "end stage" or "last year of life" or bereav* or Palliat*).ti,ab,kw. 277561

3. 1 or 2 360977

4 exp Machine Learning/ 244285

5. ("data mining" or "artificial intelligence" or "machine learning" or "deep learning" or "neural net*").ti,ab,kw. 146365

6. 4 or 5 292163

7. 3 and 6 767

8. limit 7 to (article or article in press or conference paper) 445

https://ovidsp.ovid.com/ovidweb.cgi?T=JS&NEWS=N&PAGE=main&SHAREDSEARCHID=3rPZSSUFrGNpxB…

**APA PsycInfo (OVID):**

1. exp Palliative Care/ or exp Terminal Care/ or exp Terminally Ill/ or exp Hospices/ 17587

2. ("end-of-life" or "end of life" or "end-stage" or "end stage" or "last year of life" or bereav* or Palliat*).mp. 34438

3. 1 or 2 37875

4. exp Machine Learning/ 10464

5. ("data mining" or "artificial intelligence" or "machine learning" or "deep learning" or "neural net*").mp. 57015

6. 4 or 5 57956

7. 3 and 6 55

**Scopus:**

( TITLE-ABS-KEY ( ( palliat*  OR  "terminal care"  OR   bereave  OR  hospice   OR  "end of life"  OR   "terminally ill"   OR  "end stage disease"  OR  "last year of life" ) )  AND   TITLE-ABS-KEY ( ( "machine learning"  OR  "artificial intelligence"  OR  "deep learning"  OR   "neural net*"  OR  "data mining" ) ) )  AND   ( LIMIT-TO ( DOCTYPE ,   "ar" )  OR  LIMIT-TO ( DOCTYPE ,  "cp" ) )

Document Count 373

**CINAHL EBSCO:**

S3 S1 AND S2 Search modes - Boolean/Phrase Interface - EBSCOhost Research Databases

Search Screen - Advanced Search

Database - CINAHL Plus with Full Text 119

S2 mh artificial intelligence+ or mh machine learning+ or "deep learning" or "neural net*" or "data mining" Search modes - Boolean/Phrase Interface - EBSCOhost Research Databases

Search Screen - Advanced Search

Database - CINAHL Plus with Full Text 27,087

S1 (mh Terminal Care+ OR bereave* OR hospice* OR “advanced cancer” OR “end of life” OR "end-of-life" OR "end stage disease" OR "end-stage disease" OR "last year of life" OR “terminally ill” OR palliat* OR mh Palliative Care+ OR mh palliative therapy+) Search modes - Boolean/Phrase Interface - EBSCOhost Research Databases

**Web of Science**

TOPIC: ((Palliat*   OR "terminal care"  OR bereave  OR hospice  OR "end of life"  OR "terminally ill"  OR "end stage disease"  OR "last year of life")) AND TOPIC: (("data mining"  OR "artificial intelligence"  OR "machine learning"  OR "deep learning"  OR "neural net*"))

Refined by: DOCUMENT TYPES: ( ARTICLE OR EARLY ACCESS OR PROCEEDINGS PAPER )

Timespan: All years. Indexes: SCI-EXPANDED, SSCI, A&HCI, CPCI-S, CPCI-SSH, BKCI-S, BKCI-SSH, ESCI.

Document Count 231

**Note:** All searches were originally conducted in April,2021. Record counts shown in the PRISMA diagram reflect results returned at that time. Because some databases (e.g., Scopus) only allow year-level limits and continually index new records, re-running these searches today yields higher counts than those retrieved during the original search window.

**Feb 9, 2026**

APA PsycInfo

Document Count 30

▲

Searches	Results	Type	Runtime	Actions	Annotations

1	exp Palliative Care/ or exp Terminal Care/ or exp Terminally Ill/ or exp Hospices/	22957	Advanced	0.12	Display Results

2	("end-of-life" or "end of life" or "end-stage" or "end stage" or "last year of life" or bereav* or Palliat*).mp.	44502	Advanced	2.15	Display Results

3	exp Machine Learning/	37148	Advanced	1.81	Display Results

4	("data mining" or "artificial intelligence" or "machine learning" or "deep learning" or "neural net*").mp.	90288	Advanced	2.23	Display Results

5	1 or 2	48368	Advanced	0.01	Display Results

6	3 or 4	99730	Advanced	0.02	Display Results

7	5 and 6	150	Advanced	0.02	Display Results

8	limit 7 to (full text and human and english language and yr="2021 - 2026")	30	Advanced	0.14	Display Results

**CINAHL**

| XB ( (mh Terminal Care+ OR bereave* OR hospice* OR “advanced cancer” OR “end of life” OR "end-of-life" OR "end stage disease" OR "end-stage disease" OR "last year of life" OR “terminally ill” OR palliat* OR mh Palliative Care+ OR mh palliative therapy+) ) AND XB ( (mh artificial intelligence+ or mh machine learning+ or "deep learning" or "neural net*" or "data mining" ) ) | **Limiters** - Full Text; Publication Date: 20210101-20261231  **Narrow by Language:** - english  **Search modes** - Proximity |
| --- | --- |

**Limiters** - Full Text; Publication Date: 20210101-20260228

**Narrow by Language:** - english

**Search modes** – Proximity

Document Count 14

SCOPUS

( TITLE-ABS-KEY ( ( palliat* OR "terminal care" OR bereave OR hospice OR "end of life" OR "terminally ill" OR "end stage disease" OR "last year of life" ) ) AND TITLE-ABS-KEY ( ( "machine learning" OR "artificial intelligence" OR "deep learning" OR "neural net*" OR "data mining" ) ) ) AND PUBYEAR > 2020 AND PUBYEAR < 2027 AND ( LIMIT-TO ( DOCTYPE , "ar" ) ) AND ( LIMIT-TO ( LANGUAGE , "English" ) ) AND ( LIMIT-TO ( EXACTKEYWORD , "Human" ) )

Document Count 525

**Web of Science**

((Palliat*	OR "terminal care" OR bereave OR hospice OR "end of life" OR "terminally ill" OR "end stage disease" OR "last year of life")) AND TOPIC: (("data mining" OR "artificial intelligence" OR "machine learning" OR "deep learning" OR "neural net*"))

**Refined By:Publication Years: 2025 or 2026 or 2024 or 2023 or 2022 or 2021**. Click to remove this refine from your search.**Document Types: Article**. Click to remove this refine from your search.**Languages: English**.

Document Count 94

<https://www.webofscience.com/wos/woscc/summary/c1322e87-f83b-482e-8717-e879620f0c9b-019e4a7972/3ed394cf-c1e5-4488-97c5-69d41c785aaf-019e4a699e/relevance/1>

EMBASE

Count 424

1	exp Palliative Care/ or exp Terminal Care/ or exp Terminally Ill/ or exp Hospices/	254407	Advanced	0.03	Display Results

2	("end-of-life" or "end of life" or "end-stage" or "end stage" or "last year of life" or bereav* or Palliat*).ti,ab,kw.	381957	Advanced	0.03	Display Results

3	1 or 2	495770	Advanced	0.09	Display Results

4	exp Machine Learning/	702130	Advanced	0.02	Display Results

5	("data mining" or "artificial intelligence" or "machine learning" or "deep learning" or "neural net*").ti,ab,kw.	490749	Advanced	0.02	Display Results

6	4 or 5	840948	Advanced	1.02	Display Results

7	3 and 6	3481	Advanced	0.10	Display Results

8	limit 7 to (full text and human and english language and english and yr="2021 - 2026" and article and journal)	424	Advanced	1.03	Display Results

**OVID Medline**

Count 279

1	exp Palliative Care/ or exp Terminal Care/ or exp Terminally Ill/ or exp Hospices/	120649	Advanced	0.76	Display Results

2	("end-of-life" or "end of life" or "end-stage" or "end stage" or "last year of life" or bereav* or Palliat*).ti,ab,kw.	234631	Advanced	1.31	Display Results

3	1 or 2	290216	Advanced	0.04	Display Results

4	exp Machine Learning/	115026	Advanced	0.06	Display Results

5	("data mining" or "artificial intelligence" or "machine learning" or "deep learning" or "neural net*").ti,ab,kw.	415372	Advanced	1.24	Display Results

6	4 or 5	430436	Advanced	0.07	Display Results

7	3 and 6	1334	Advanced	0.07	Display Results

8	limit 7 to (english language and full text and humans and yr="2021 - 2026" and english and journal article)	279	Advanced	0.41	Display Results
